# Supplementary material for: Novel Adaption of the SARC-F Score to Classify Pediatric Hemato-Oncology Patients with Functional Sarcopenia
Source: Cancers (Basel). 2023 Jan 3;15(1):320. doi: 10.3390/cancers15010320 (PMC9818846; doi:10.3390/cancers15010320)
Supplement: Supplementary file 1 [file cancers-15-00320-s001.zip › Figure S1.pdf]

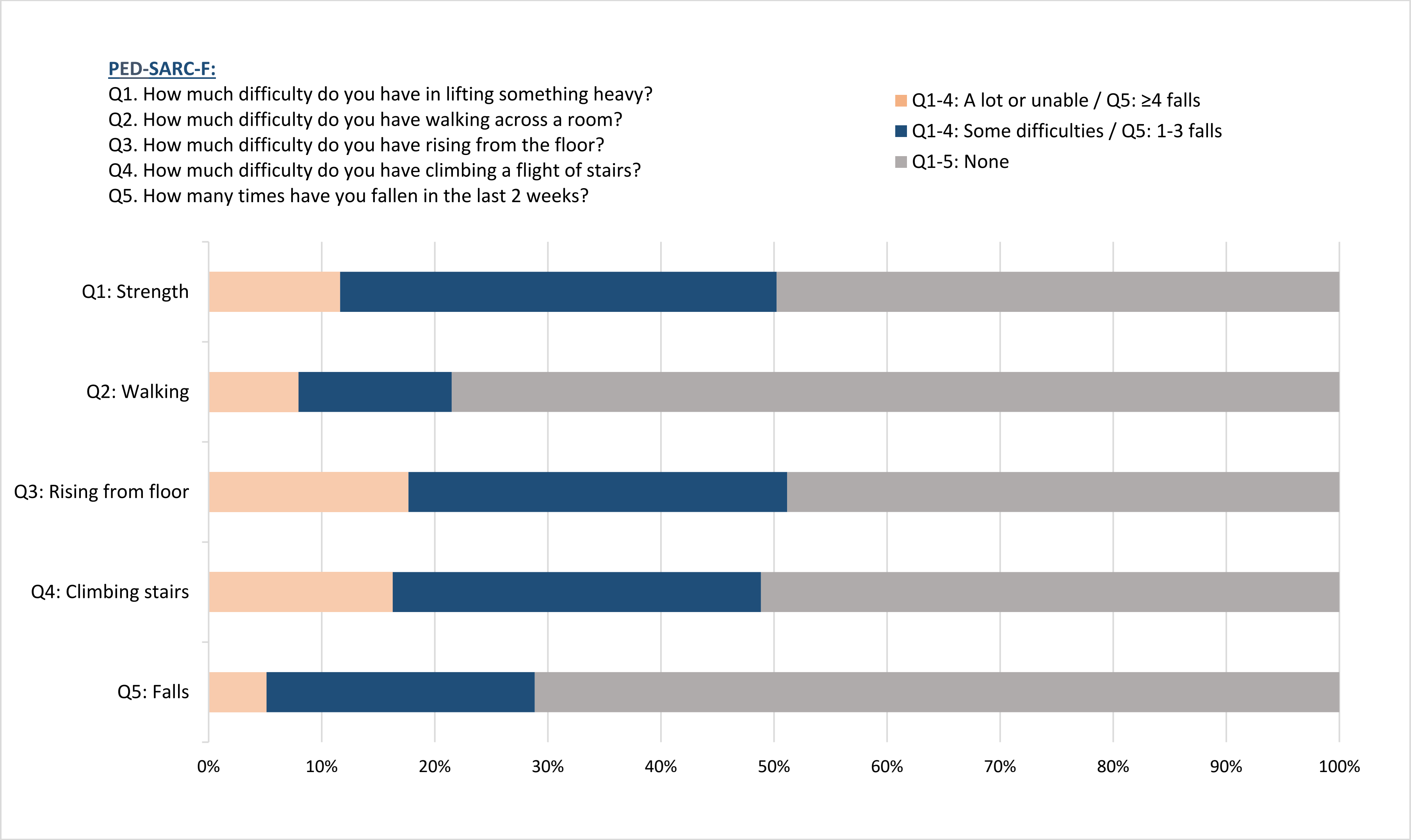

Figure S1. The pediatric SARC-F (PED-SARC-F) and the results on the individual questions
