# Supplementary material for: Novel Adaption of the SARC-F Score to Classify Pediatric Hemato-Oncology Patients with Functional Sarcopenia
Source: Cancers (Basel). 2023 Jan 3;15(1):320. doi: 10.3390/cancers15010320 (PMC9818846; doi:10.3390/cancers15010320)
Supplement: Supplementary file 1 [file cancers-15-00320-s001.zip › Table S1.pdf]

Table S1. Description of patients with ≥ 1 physiotherapy assessment(s)

			At time of physiotherapy assessment		
**Patient**	**Tumor type**	**Assessment no.**	**Age, years**	**Treatment phase**	**PED- SARC-F score**
1	AML	1	16.4	Pre SCT	4
		2	17.3	10 months post SCT	4
2	BPDCN	1	15.4	3 months post SCT	6
		2	15.9	9 months post SCT	3
3	SAA	1	16.4	7 months post SCT	2
		2	16.8	12 months post SCT	0
4	Pre B-ALL	1	3.3	First week of therapy	2
		2	3.6	End of induction phase	8
5	Pre B-ALL	1	3.8	Consolidation phase	9
		2	5.4	Pre SCT	3
		3	5.6	3 months post SCT	2
6	MDS	1	14.4	Pre SCT	2
		2	14.8	3 months post SCT	0
7	Pre B-ALL	1	3.8	Pre SCT	7
		2	4.3	5 months post SCT	4
8	Pre B-ALL	1	7.5	Induction phase	5
		2	8	Consolidation phase	3
9	T-ALL	1	16.7	Induction phase	3
		2	17.4	Maintenance phase	0
10	BCR-ABL-like ALL	1	17.3	3 months post SCT	3
		2	17.5	5 months post SCT	3
11	Pre B-ALL	1	15.4	Pre SCT	0
		2	15.7	3 months post SCT	1
12	FA	1	12.5	3 months post SCT	2
		2	12.8	6 months post SCT	0
13	T-ALL	1	9.8	Pre SCT	5
		2	10.3	3 months post SCT	3
14	Pre B-ALL	1	15.5	Induction phase	5
		2	16.5	Consolidation phase	3
15	Pre B-ALL	1	16.6	Induction phase	5
		2	17.7	Maintenance phase	0
16	C-ALL	1	16.5	Induction phase	4
		2	17.4	Maintenance phase	2
		3	17.6	At therapy cessation	1
17	FA	1	7.5	Pre SCT	0
		2	7.8	3 months post SCT	0
18	C-ALL	1	12.7	3 months post SCT	6
		2	12.9	7 months post SCT	3
19	AML	1	17.3	Pre SCT	0
		2	17.5	3 months post SCT	0
20	AML	1	14.5	3 months post SCT	2
		2	14.9	8 months post SCT	0
21	T-ALL	1	11.4	Pre SCT	1
		2	11.8	3 months post SCT	3
22	Pre B-ALL	1	18.2	Maintenance phase	3

		2	18.5	Maintenance phase	1
		3	19.5	6 weeks post therapy cessation	1
23	FA	1	8.5	Pre SCT	0
		2	8.9	3 months post SCT	0
24	Pre B-ALL	1	8.1	Maintenance phase	7
		2	9.9	3 months post therapy cessation	0
25	Pre B-ALL	1	12	Maintenance phase	4
		2	12.6	Pre SCT	0
		3	13	4 months post SCT	0
26	MDS	1	13.3	Pre SCT	0
		2	14.1	6 months post SCT	0
27	Pre B-ALL	1	7.5	Induction phase	2
		2	8.8	Maintenance phase	0
28	AML	1	15.1	3 months post SCT	3
		2	15.3	6 months post SCT	0
29	FA	1	13.9	Pre SCT	0
		2	14.3	3 months post SCT	0
30	FA	1	10.4	Pre SCT	2
		2	10.8	3 months post SCT	1
		3	11.3	9 months post SCT	0
31	Pre B-ALL	1	17	Maintenance phase	6
		2	17.4	Maintenance phase	0
32	Pre B-ALL	1	11.2	Maintenance phase	3
		2	11.5	Maintenance phase	5
33	Pre B-ALL	1	17.9	Induction phase	5
		2	18.4	Maintenance phase	4
34	NHL	1	11.7	Pre SCT	0
		2	12.2	6 months post SCT	9
		3	12.5	9 months post SCT	5
35	AML	1	14.6	Pre SCT	3
		2	14.9	3 months post SCT	3
36	Pre B-ALL	1	17	Induction phase	7
		2	18.8	Maintenance phase	3
		3	19.1	3 months post therapy cessation	0
37	AML	1	11.3	Intensive chemotherapy	3
		2	11.8	3 months post SCT	0
38	Pre B-ALL	1	8.3	Induction phase	6
		2	8.9	Maintenance phase	6
39	CML	1	11.8	Pre SCT	6
		2	12.1	3 months post SCT	2
40	ALCL	1	13.7	Pre SCT	2
		2	14.1	3 months post SCT	1
41	CML	1	15.6	Pre SCT	3
		2	16.1	3 months post SCT	2
Abbreviations:					

AML = acute myeloid leukemia, BPDCN = blastic plasmacytoid dendritic cell neoplasm, SAA = severe aplastic anemia, ALL = acute lymphoblastic leukemia, MDS = myelodysplastic syndrome, FA = Fanconi anemia, NHL = Non-hodgkin lymphoma, ALCL = anaplastic large cell lymphoma
