# Supplementary material for: Novel Adaption of the SARC-F Score to Classify Pediatric Hemato-Oncology Patients with Functional Sarcopenia
Source: Cancers (Basel). 2023 Jan 3;15(1):320. doi: 10.3390/cancers15010320 (PMC9818846; doi:10.3390/cancers15010320)
Supplement: Supplementary file 1 [file cancers-15-00320-s001.zip › Table S2.pdf]

Table S2. Characteristics of individual patients at their first physiotherapy assessment (n=167)		
	**No.**	**%**
Sex		
Boy	105	62.9
Girl	62	37.1
Type of hematological disease		
Acute lymphoblastic leukemia	102	61
Acute myeloid leukemia	18	10.8
Chronic myeloid leukemia	4	2.4
Hodgkin lymphoma	8	4.8
Non-hodgkin lymphoma	13	7.8
Myelodysplastic syndrome	6	3.6
Fanconi anemia	8	4.8
Aplastic anemia	2	1.2
Other*	6	3.6
Treatment phase		
Intensive chemotherapy	51	30.5
Maintenance chemotherapy	46	27.5
1-12 months after chemotherapy cessation	16	9.6
Pre SCT conditioning phase	29	17.4
3-12 months post SCT	25	15
Assessment performed during		
Clinical admission	34	20.4
Daycare admission / Outpatient clinic visit	133	79.6
Body Mass Index, categories		
Underweight	11	6.6
Normal Weight	114	68.2
Overweight	31	18.6
Obesity	11	6.6
	**Mean**	**Median [IQR]**
Age, years	11.7	12.7 [7.5 to 15.6]
Height, SDS	-0.27	-0.22 [-1 to 0.47]
Weight, SDS	0.30	0.20 [-0.8 to 1.2]
Body Mass Index, SDS	0.36	0.32 [-0.8 to 1.5]

Abbreviations: SCT = stem cell transplantation, IQR = interquartile range, SDS = standard deviation score
*Blastic plasma cytoid dendritic cell neoplasm (n = 1), common variable immunodeficiency (n = 1), de novo acute promyelocytic leukemia (n =1), Diamond-Blackfan anemia (n = 1), Langerhans cell histiocytosis (n = 1), Paroxysmal nocturnal hemoglobinuria (n = 1)
