# Supplementary material for: Novel Adaption of the SARC-F Score to Classify Pediatric Hemato-Oncology Patients with Functional Sarcopenia
Source: Cancers (Basel). 2023 Jan 3;15(1):320. doi: 10.3390/cancers15010320 (PMC9818846; doi:10.3390/cancers15010320)
Supplement: Supplementary file 1 [file cancers-15-00320-s001.zip › Table S3.pdf]

Table S3. Diagnostic accuracy for the sarcopenia phenotype at different cut-off points of the PED-SARC-F			
	Functional Sarcopenia		
	Yes	No	
PED-SARC-F score			
≥1	44	96	PPV: 0.31
<1	2	73	NPV: 0.97
	Sensitivity: 0.96	Specificity: 0.43	
AUC: 0.69 (95% CI: 0.65 to 0.74)			
PED-SARC-F score			
≥2	43	77	PPV: 0.36
<2	3	92	NPV: 0.97
	Sensitivity: 0.93	Specificity: 0.54	
Diagnostic accuracy: 0.74 (95% CI: 0.69 to 0.79)			
PED-SARC-F score			
≥3	42	52	PPV: 0.45
<3	4	117	NPV: 0.97
	Sensitivity: 0.91	Specificity: 0.69	
Diagnostic accuracy: 0.80 (95% CI: 0.75 to 0.86)			
PED-SARC-F score			
≥4	36	29	PPV: 0.55
<4	10	140	NPV: 0.93
	Sensitivity: 0.78	Specificity: 0.83	
Diagnostic accuracy: 0.81 (95% CI: 0.74 to 0.87)			
PED-SARC-F score			
≥5	34	16	PPV: 0.68
<5	12	153	NPV: 0.93
	Sensitivity: 0.74	Specificity: 0.91	
Diagnostic accuracy: 0.82 (95% CI: 0.75 to 0.89)			
PED-SARC-F score			
≥6	27	6	PPV: 0.82
<6	19	163	NPV: 0.90
	Sensitivity: 0.59	Specificity: 0.96	
Diagnostic accuracy: 0.78 (95% CI: 0.7 to 0.85)			
PED-SARC-F score			
≥7	21	3	PPV: 0.88
<7	25	166	NPV: 0.87
	Sensitivity: 0.46	Specificity: 0.98	
Diagnostic accuracy: 0.72 (95% CI: 0.65 to 0.79)			
PED-SARC-F score			
≥8	16	1	PPV: 1
<8	30	168	NPV: 0.82
	Sensitivity: 0.22	Specificity: 1	
Diagnostic accuracy: 0.67 (95% CI: 0.6 to 0.74)			
PED-SARC-F score			
≥9	10	0	PPV: 0.7
<9	36	169	NPV: 0.82
	Sensitivity: 0.16	Specificity: 0.98	
Diagnostic accuracy: 0.61 (95% CI: 0.55 to 0.67)			
PED-SARC-F score			
≥10	3	0	PPV: 1
<10	43	169	NPV: 0.8
	Sensitivity: 0.07	Specificity: 1	
Diagnostic accuracy: 0.53 (95% CI: 0.5 to 0.57)			

Abbreviations: AUC = area under the curve PPV = positive predicted value, NPV = negative predicted value, CI = Confidence interval
